# Supplementary material for: From sequence to enzyme mechanism using multi-label machine learning
Source: BMC Bioinformatics. 2014 May 19;15:150. doi: 10.1186/1471-2105-15-150 (PMC4229970; doi:10.1186/1471-2105-15-150)
Supplement: Additional file 2 — Java code of ml2db. Additional file ml2db_code.tar.gz contains the Java source code to run the multi-label machine learning experiments and save the results to database. The code’s Javadoc is included. [file 1471-2105-15-150-S2.zip › additional file 2/ml2db/utils/doc/uk/ac/ed/inf/utils/collectionutils/CollectionUtils.html]

CollectionUtils


---


|  |  |  |
| --- | --- | --- |
| **Overview** \| **Package** \| **Class** \| **Use** \| **Tree** \| **Deprecated** \| **Index** \| **Help** | |  |
| **PREV CLASS**   **NEXT CLASS** | **FRAMES**    **NO FRAMES**     **All Classes** |  |
| SUMMARY: NESTED \| FIELD \| CONSTR \| METHOD | DETAIL: FIELD \| CONSTR \| METHOD |  |


---


## uk.ac.ed.inf.utils.collectionutils Class CollectionUtils

```
java.lang.Object
  uk.ac.ed.inf.utils.collectionutils.CollectionUtils
```

---

``` public class CollectionUtils extends java.lang.Object ```

Class

**Version:**
:   1 Jul 2008

**Author:**
:   Luna De Ferrari luna.deferrari-at-ed.ac.uk

---

**Constructor Summary**

`CollectionUtils()`


| **Method Summary** | |
| --- | --- |
| `static int` | `countValues(java.util.Collection collection, java.lang.Object value)`             Counts the occurrences of a given value in a collection (using the 'equals' method) |
| `static java.util.Vector<java.lang.String>` | `fromArrayListToVector(java.util.ArrayList<java.lang.String> list)` |
| `static java.util.Vector<java.lang.String>` | `fromBigSetToVector(java.util.Set<java.lang.String> set)`             Deletes each set entry after writing it to vector, to limit memory use. |
| `static java.util.Vector<java.lang.String>` | `fromSetToVector(java.util.Set<java.lang.String> set)` |

**Methods inherited from class java.lang.Object**

`equals, getClass, hashCode, notify, notifyAll, toString, wait, wait, wait`

**Constructor Detail**

### CollectionUtils

```
public CollectionUtils()
```


**Method Detail**

### countValues

```
public static int countValues(java.util.Collection collection,
                              java.lang.Object value)
```

:   Counts the occurrences of a given value in a collection (using the
    'equals' method)

    :   **Parameters:**: `collection` - a collection: `value` - an object **Returns:**: the count of the object occurrences in the collection

---


### fromArrayListToVector

```
public static java.util.Vector<java.lang.String> fromArrayListToVector(java.util.ArrayList<java.lang.String> list)
```

---


### fromBigSetToVector

```
public static java.util.Vector<java.lang.String> fromBigSetToVector(java.util.Set<java.lang.String> set)
```

:   Deletes each set entry after writing it to vector, to limit memory use.

    :   **Parameters:**: `a` - big set **Returns:**: a vector of the set elements

---


### fromSetToVector

```
public static java.util.Vector<java.lang.String> fromSetToVector(java.util.Set<java.lang.String> set)
```


---


|  |  |  |
| --- | --- | --- |
| **Overview** \| **Package** \| **Class** \| **Use** \| **Tree** \| **Deprecated** \| **Index** \| **Help** | |  |
| **PREV CLASS**   **NEXT CLASS** | **FRAMES**    **NO FRAMES**     **All Classes** |  |
| SUMMARY: NESTED \| FIELD \| CONSTR \| METHOD | DETAIL: FIELD \| CONSTR \| METHOD |  |


---
